# The hybrid nanosystem for the identification and magnetic hyperthermia immunotherapy of metastatic sentinel lymph nodes as a multifunctional theranostic agent

**DOI:** 10.3389/fbioe.2024.1445829

**Published:** 2024-07-29

**Authors:** Qiaoxi Qin, Qin Zhang, Pan Li, Ronghui Wang, Ying Liu, Ruxi Deng, Juanmin Zhang, Quanyu Nie, Hong Zhou, Yang Zhou

**Affiliations:** ^1^ Department of Ultrasound, The Third People’s Hospital of Chengdu, The Affiliated Hospital of Southwest Jiaotong University, Chengdu, China; ^2^ Department of Radiology, Chongqing Hospital of Traditional Chinese Medicine, Chongqing, China; ^3^ Department of Ultrasound, The Second Affiliated Hospital of Chongqing Medical University, Chongqing, China; ^4^ Department of Ultrasound, Ruijin Hospital, Shanghai Jiaotong University School of Medicine, Shanghai, China

**Keywords:** sentinel lymph nodes, lymph node metastasis, contrast-enhanced ultrasound, magnetic hyperthermia, immunotherapy

## Abstract

Lymphatic metastasis is the main cause of early-stage tumor spread, making the identification and therapy of metastatic sentinel lymph nodes (SLNs) are highly desirable in clinic. Currently, suspected malignant SLNs typically undergo a series of independent operations in clinical practice, including imaging, staining, sentinel lymph node biopsy (SLNB) and lymph node dissection (LND), which brings inconvenience to diagnosis and treatment, and may cause postoperative complications for patients. Moreover, the ordinary removal of tumor-draining lymph nodes (TDLNs) may do harm to systemic immunity required for tumor eradication. Hence, we utilized the hybrid nanosystem (SPIOs + RPPs) we constructed before for the integrated staining, ultrasound imaging, and therapy of metastatic SLNs. In this study, SPIOs + RPPs could migrate into SLNs successfully to stain them black for easy visual identification. Beyond staining, the hybrid nanosystem could realize contrast enhanced ultrasound (CEUS) imaging in SLNs. Meanwhile, it could inhibit cancer cells to lower the tumor burden and reverse immune-suppressive microenvironment of metastatic SLNs effectively via magnetic hyperthermia immunotherapy in VX2 tumor-bearing rabbits with popliteal fossa lymph node metastasis. These findings indicate that SPIOs + RPPs is a potential multifunctional theranostic agent for detection and therapy of lymphatic metastasis.

## 1 Introduction

Malignant tumors remain a threat to public health as a leading cause of mortality worldwide, with over 90% of cancer-related deaths attributed to metastatic disease rather than the primary tumors (Chaffer and Weinberg, 2011; Castaneda et al., 2022). At the early stage of tumor metastasis, lymphatic metastasis is the predominant way of cancer cells spread. Sentinel lymph nodes (SLNs) play an important role in this process, receiving lymphatic drainage from the primary malignant tumor firstly and may be the initial site where metastasis forms (Liang et al., 2014). Consequently, the early identification and interventions of metastatic SLNs is of great significance in clinical practice.

Sentinel lymph node biopsy (SLNB) is the standard diagnostic procedure for assessing regional lymph node metastasis in clinic setting currently (Cykowska et al., 2020). Accurate SLNs location is the most important prerequisite for SLNB (Lin et al., 2022), and the current “gold standard” to localize the SLNs is the combined technique by injecting radiotracer 99 mTc alongside blue dye (Thill et al., 2014). However, this combined technique is not readily accessible as it is equipment-demanding, expensive and radioactive (Myagmarjalbuu et al., 2013). Additionally, the efficacy of blue dye lymphatic mapping is restricted by tissue penetration and staining artifacts, which may interfere surgical visualization (Liang et al., 2014; Yang et al., 2017). Therefore, there is a growing demand for a straightforward, non-radioactive, and efficient approach for SLNs detection. Since Mattrey RF et al. firstly applied contrast-enhanced ultrasound (CEUS) on SLN identification in breast cancer patients, it has been constantly studied because of its ease of use, no radiation and cost-effectiveness (Mattrey et al., 2002; Zhao et al., 2018; Lahtinen et al., 2022; Chu et al., 2023). Increasing researches have demonstrated that microbubbles and the enhanced patterns on CEUS may be helpful in recognizing metastasizing SLNs (Xie et al., 2015). However, conventional ultrasound contrast agents, such as microbubbles, are often too large (200–500 nm) to enter the endothelial gap in lymphatic capillaries (Lin et al., 2022). Microbubbles also suffer from instability, and short circulation time (Qin et al., 2023). In recent years, the imaging function of phase-transition nanodroplets has been explored extensively (Wilson et al., 2012; Huang et al., 2017). Poly (lactic-co-glycolic acid, PLGA) liquid‒gas phase-transition nanodroplets, which can transform into microbubbles form nanodroplets upon the external stimulation (ultrasonic wave or thermal stimuli), may be an ideal lymphatic tracer. Their nanoscale size allows for easier passage through endothelial gaps, and they exhibit enhanced stability and prolonged retention in circulatory system (Qi et al., 2022).

While CEUS can help locate lymph nodes, a visible dye is essential to mark SLNs to facilitate subsequent operations. Compared with methylene blue (one of the most commonly used blue dye for marking SLNs in clinical practice), magnetic nanoparticles (MNPs) demonstrate superior long-term stability and minimal peripheral tissue staining. Additionally, MNPs exhibit potential in replacing the combined technique in SLNB due to some of their shared characteristics. Firstly, they can be externally detected in preincision, secondly, they are of similar dimensions to the radiotracer colloid, and thirdly, their brown-black color acts as a visual marker (Ahmed and Douek, 2013). Moreover, the superparamagnetic behavior of supermagnetic iron oxide nanoparticles (SPIOs) makes them ideal for SLNB. SPIOs do not agglomerate when being transported via lymph in the absence of an alternating magnetic field (AMF), while under an AMF, their collective moment can be sensed external to the body (Harnan et al., 2011).

In clinical oncology, SLNs usually undergo a series of procedures in clinical settings, including imaging, staining, biopsy and lymph node dissection (LND), bringing inconvenience to the diagnosis and treatment process and incurring additional time and economic costs. Moreover, LND could be challenging due to severe tissue invasion and convert anatomical location of lymph nodes, and it may cause some postoperative complications such as lymphedema and sensory disturbances, impacting the life quality of patients (Fish et al., 2020; Wu et al., 2022). Furthermore, tumor-draining lymph nodes (TDLNs) play an important role in immune surveillance and tumor rejection as the main anti-tumor immune organ (Naxerova et al., 2017; Pereira et al., 2018), and the ordinary removal of TDLNs may do harm to systemic immunity required for tumor eradication. These shortcomings and controversy highlight a demand for a convenient and non-invasive theranostic modality for the identification and treatment of metastatic SLNs.

Hyperthermia therapy (HTT) has emerged as a potential strategy against tumors on account of its unique advantages compared with conventional surgery methodologies, such as minimal invasiveness, fewer side effects and improved tumor specificity, etc (Okuno et al., 2013; Liang et al., 2014; Cai et al., 2016; Shi et al., 2018). In recent years, the application of photothermal therapy (PTT) ablation on SLNs has been suggested by several reports for tumor eradication (Cai et al., 2016; Wu et al., 2022; Deng et al., 2023). Magnetic hyperthermia (MHT) exhibits unparalleled superiority over PTT for its penetration depth without tissue attenuation, owing to the magnetic responsiveness of MNPs under an AMF (Thiesen and Jordan, 2008; Moros et al., 2019). Although researches on MHT for metastatic lymph nodes are currently limited, its potential warrants further investigation. Fortunately, the multifunctionality of SPIOs renders it suitable for theranostic applications in SLN-mediated metastasis. In addition to SLNs mapping, SPIOs could be applied for MHT, where the magnetic-responsive heat could not only be used for the ablation of tumor cells, but also could activate the phase-transition nanodroplets for SLNs CEUS.

The traditional dogma that the dissection of clinically positive lymph nodes results in improved survival has been eroded step by step with the deepening researches and clinical evidence (Morton et al., 2006; Morton et al., 2014; Faries et al., 2017; Giuliano et al., 2017). Immunotherapy has been considered as the most promising approach to defeat cancer, and the role of TDLNs in immunotherapy has received increasing attention. However, during the progression of tumor, the immune microenvironment of TDLNs could be disrupted by the invasion of cancer cells, including the variation of immune cells composition, impairment of antigen presentation function, and alteration of MHC class I-molecules or immunosuppressive ligands in tumor cells, shifting the original activity of TDLNs as organs of anti-tumor immune surveillance to an immune escape state that sustains the growth of the tumor (van Pul et al., 2019; Molodtsov et al., 2021; Rahim et al., 2023). As the most important APC, dendritic cells (DCs) in SLN have been demonstrated to be more immune suppressed than DCs in further downstream located TDLN in early pioneering studies (Cochran et al., 2001; Matsuura et al., 2006; van Pul et al., 2019). This observation suggested DCs to be a prime target of immune suppression, consistent with their pivotal role in initiating T-cell-mediated anti-tumor immunity. Resiquimod (R848), a potent agonist of Toll-like receptor 7 and 8 (TLR-7/8), can improve the body’s immune responses to antigens and activate DCs to evoke the immune cascade, which is highly reliable for targeted regulation of DCs.

**FIGURE 1:**
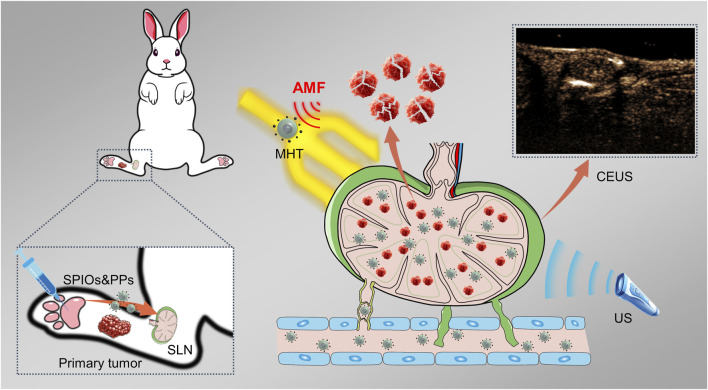
Schematic illustration of the identification and magnetic hyperthermia immunotherapy of metastatic sentinel lymph nodes using SPIOs + RPPs.

In our recent study, we developed a hybrid nanosystem (SPIOs + RPPs) consisting of SPIOs and PLGA liquid‒gas phase-transition nanodroplets, with R848 as an immunoadjuvant and perfluoropentane (PFP) as a phase transition agent, named R848-PFP@PLGA(RPPs). SPIOs + RPPs has been proven to achieve tumor ultrasound imaging, activate anti-tumor immunity and realize enhanced mild magnetic hyperthermia (MMHT) for inhibiting tumor proliferation and metastasis (Qin et al., 2023). Based on the aforementioned ideas and our previous findings, we continued to explore the potential of SPIOs + RPPs for visual detection of SLNs, and the ability of reducing tumor burden on metastatic lymph nodes while preserving its anti-tumor immunity, and preliminarily discuss the anti-tumor function and mechanism of SLNs in immunotherapy, as illustrated in Figure 1. Our work presents that SPIOs + RPPs integrates the function of staining, contrast enhanced ultrasound (CEUS) imaging and therapy, effectively simplifying the diagnostic and treatment process. And we applied magnetic hyperthermia immunotherapy on metastatic SLNs for the first time, effectively inhibited cancer cells to reduce the tumor burden, and activated immune cells to reverse immune-suppressive microenvironment in metastatic SLNs.

This study demonstrates the potential of SPIOs + RPPs for theranostic application in lymphatic metastasis, which may provide an alternative strategy for preventing tumor metastasis amenable for clinical use.

## 2 Experimental section

### 2.1 Materials

The materials used for preparing the hybrid nanosystem is the same as mentioned in our previous article (Qin et al., 2023). Methylene blue was purchased from Beyotime Biotechnology (China). Antibodies to cell surface markers for immunofluorescence assay of TUNEL and PCNA were purchased from BioLegend, Inc. (United States).

### 2.2 Synthesis of the hybrid nanosystem

The details of the preparation of SPIOs + RPPs as described previously (Qin et al., 2023). SPIOs were prepared by co-precipitation method, and RPPs were synthesized through a typical double-emulsion process. Resuspend SPIOs and RPPs proportionally in saline to obtain the hybrid nanosystem dispersion. 1 mL dispersion containing 1.2 mg Fe and 8 mg RPPs.

### 2.3 Animal models

New Zealand white rabbits (weight range of 2.5–4.0 kg) were purchased from Animal Center of Chongqing Medical University. Tumor-bearing rabbits with a VX2 tumor in liver were obtained from the laboratory of Ultrasound Engineering Institute of Chongqing Medical University. The rabbits were anesthetized by intravenous injection with 3% pentobarbital solution (1 mL/kg), and the abdomens of the rabbits were depilated with hair removal cream. In the supine position, the rabbits were routinely sterilized. Then, the VX2 liver tumors were excised and soaked in sterile PBS. Next, the tumor was cut with eye scissors into pieces of approximately 1.0 mm^3^ under sterile conditions. Subsequently, after the similar anesthesia, depilating and disinfecting, a hole was created reaching to subskin with 12G syringe needle in the left lateral hind legs of rabbits, then one piece of tumor tissue was implanted into the hole, and the wound was sutured after hemostasis.

### 2.4 *In vivo* SLNs staining of the hybrid nanosystem

When popliteal fossa lymph nodes could be palpated about 3 weeks after tumor inoculation, rabbits were anesthetized, depilated and fixed in the side-lying position.

SPIOs and SPIOs + RPPs dispersed respectively in 2 mL PBS (Fe: 2.40 mg, RPPs:8 mg) were prepared for percutaneous injection via foot pad of the hind legs, and 2 mL saline and methylene blue were injected as control. After 1 h, the popliteal fossa lymph nodes were dissected to observe their appearance changes.

### 2.5 *In vivo* SLNs ultrasound imaging of the hybrid nanosystem

An ultrasound imaging system (Vevo LAZR, Canada) was used to evaluate the MDV capability and the SLNs ultrasound imaging performance of SPIOs + RPPs. The US imaging mode parameters were fixed (frequency: 18 MHz; power: 4%; contrast gain: 43 dB; 2D gain: 22 dB; dynamic range: 35 dB). When popliteal fossa lymph nodes could be palpated, the rabbits were separated into four groups with randomization (n = 3).

After anesthetization, depilation and fixed in the side-lying position, these VX2 tumor bearing rabbits received a percutaneous injection of 2 mL saline, SPIOs, RPPs, and SPIOs + RPPs (Fe:1.2 mg mL^−1^, RPPs:4 mg mL^−1^) via foot pad of the hind legs. The US images of tumor at both B-mode and CEUS mode were recorded immediately after AMF irradiation (500 kHz; 45 A; 10 min). The analysis of the echo intensity of the ROI was acquired through DFY software (Chongqing Medical University, Chongqing, China).

### 2.6 *In vivo* MHT ablation of SLNs

The experimental procedure is similar to *in vivo* imaging part mentioned above. In short, for AMF-free groups, rabbits were just injected with 2 mL SPIOs, and SPIOs + RPPs (Fe: 1.2 mg mL^−1^, RPPs:4 mg mL^−1^) without AMF irradiation. And for the AMF stimulation groups, 1 h after injection with 2 mL SPIOs and SPIOs + RPPs, rabbits were irradiated with AMF (45 A, 500 kHz) for 10 min and repeated three times. The temperature in SLN was monitored by a thermal infrared camera during exposure to the AMF.

### 2.7 Immunofluorescence assay (IFA) of dissected lymph nodes

The rabbits were sacrificed at day 3 after different treatments and the popliteal fossa lymph nodes were excised for pathological examination on H&E, TUNEL, PCNA, Tregs and CTLs staining. Lymph nodes were mounted with OCT compound. 5 μm slices were separated by the microtone, and slices underwent the standard procedures of blocking–washing–incubating–staining. We used ImageJ software (National Institutes of Health, United States) to measure the fluorescence intensity of specific protein for semi quantitative analysis of TUNEL, PCNA, Tregs and CTLs. Select color channels tool and then split channels, and set measurement, and select the region of interest for fluorescence intensity measurement. Finally, perform statistical analysis on measured values (n = 3).

### 2.8 Assessment of DC maturation in SLNs

To study the activation of DCs in SLNs, 3 days after diverse treatments, the popliteal fossa lymph nodes in five groups were collected. The maturity of DCs in the lymph nodes was then examined by flow cytometry after immunofluorescence staining anti-CD11c FITC, anti-CD86 PE and anti-CD80 PC5.5 (BioLegend, United States) antibody according to the procedure of the manufacturer and then sorted by flow cytometry.

## 3 Results and discussion

### 3.1 The SLNs staining by SPIOs + RPPs

**FIGURE 2:**
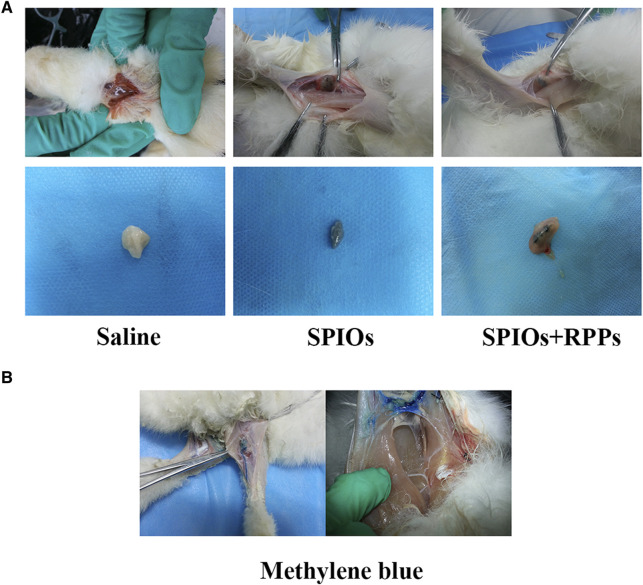
The function of the hybrid nanosystem in SLNs staining. **(A)** Staining of popliteal fossa lymph nodes *in vivo* after the injection of saline, SPIOs, and SPIOs + RPPs (upper row). Dissected lymph nodes stained in saline, SPIOs, SPIOs + RPPs (lower row). **(B)** The methylene blue is draining through the lymphatic vessel (left). Expand scope of blue stained lymph node area (right).

Due to the structural and functional characteristics of the lymphatic system, interstitial injection of dye or contrast agents remains the most promising administration route towards LNs at present (Proulx et al., 2013). In order to evaluate the staining function of the hybrid nanosystem in lymph nodes, we injected the contrast agents via the paw pad of rabbits’ hind legs. As shown in Figure 2A, the popliteal fossa lymph nodes receiving SPIOs alone and SPIOs + RPPs were stained black (the color of SPIOs), while the negative control group exhibited no signs of staining. We also prepared a positive control group using methylene blue (Figure 2B). Draining through the lymphatic vessels, methylene blue reached the lymph node area and caused non-specific blue staining of the surrounding tissues, interfering the visibility of the surgery area. In contrast, the areas stained by the hybrid nanosystem presented a clear and easily distinguishable view. The results demonstrates that SPIOs + RPPs could be absorbed into the lymphatic vessels to facilitate the staining of lymph nodes, and the black-stained lymph nodes can be easily distinguished from the surrounding tissue, which is in favor of intraoperative manipulation. Researchers have reported that the size and charge of the nanoparticles could influence their transport to the lymph nodes, specifically, the smaller nanoparticles (>10 nm) are taken up preferentially via the lymphatic system and anionically charged nanoparticles show enhanced retention in the lymph nodes (Zou et al., 2019; Nakamura et al., 2020; Nakamura and Harashima, 2020). The adequate absorption and staining may be attributed to the nano-size and negative charge of the hybrid nanosystem (The characterization of the hybrid nanosystem is presented in our recent study (Qin et al., 2023)).

### 3.2 *In vivo* magnetic hyperthermia performance of SPIOs + RPPs

**FIGURE 3:**
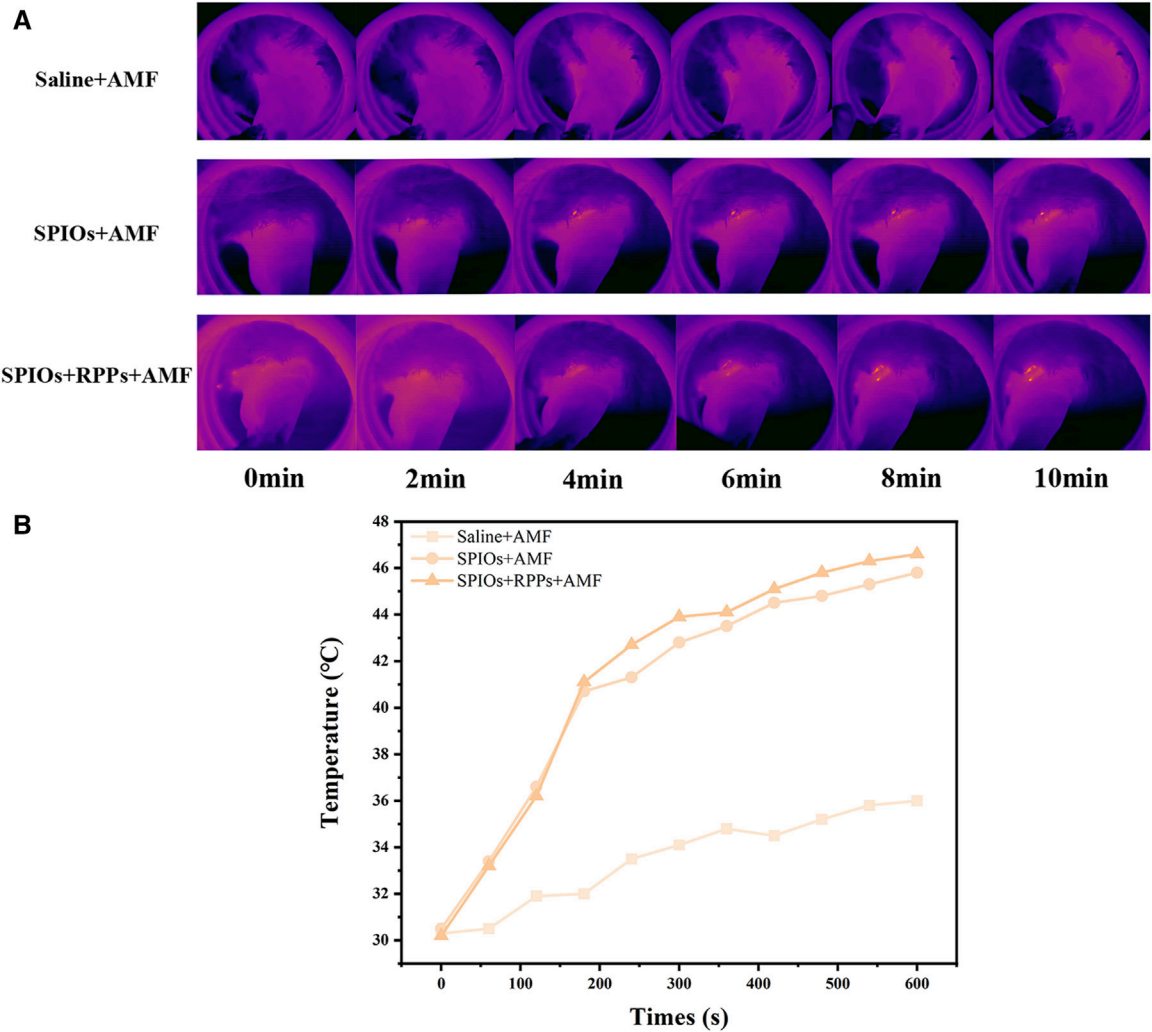
*In vivo* magnetic hyperthermia performance of hybrid nanosystem. **(A)** Infrared thermal images of VX2 tumor-bearing rabbits under different treatment groups. **(B)** Quantitative temperature–time curves based on IR thermal imaging data in **(A)**.

After the enrichment of the hybrid nanosystem in metastatic SLNs, the local temperature would rise under AMF irradiation. The popliteal fossa lymph nodes were exposed to AMF irradiation 1 h after the subcutaneous injection, and the temperature changes were monitored by an infrared thermal imaging system. As shown in Figure 3A, the rabbits’ popliteal fossa area in the SPIOs + AMF and SPIOs + RPPs + AMF group exhibited bright orange-red spots, gradually intensifying over time, but there was no obvious change in the control group in the absence of SPIOs. According to the temperature rise curves recorded by the infrared thermal imager (Figure 3B), a significant temperature increase was observed in the groups with concurrent AMF irradiation and SPIOs components compared with the control group. The temperature in the SPIOs + AMF and SPIOs + RPPs + AMF groups rapidly increased to ∼41°C within 200 s, and then steadily rose to ∼46°C over the next 400 s.The results demonstrate the superior magnetic heating efficiency of SPIOs, not compromised by the introduction of RPPs, further validating the excellent *in vivo* magnetic hyperthermia performance of the hybrid nanosystem.

### 3.3 The SLNs ultrasound imaging of SPIOs + RPPs

**FIGURE 4:**
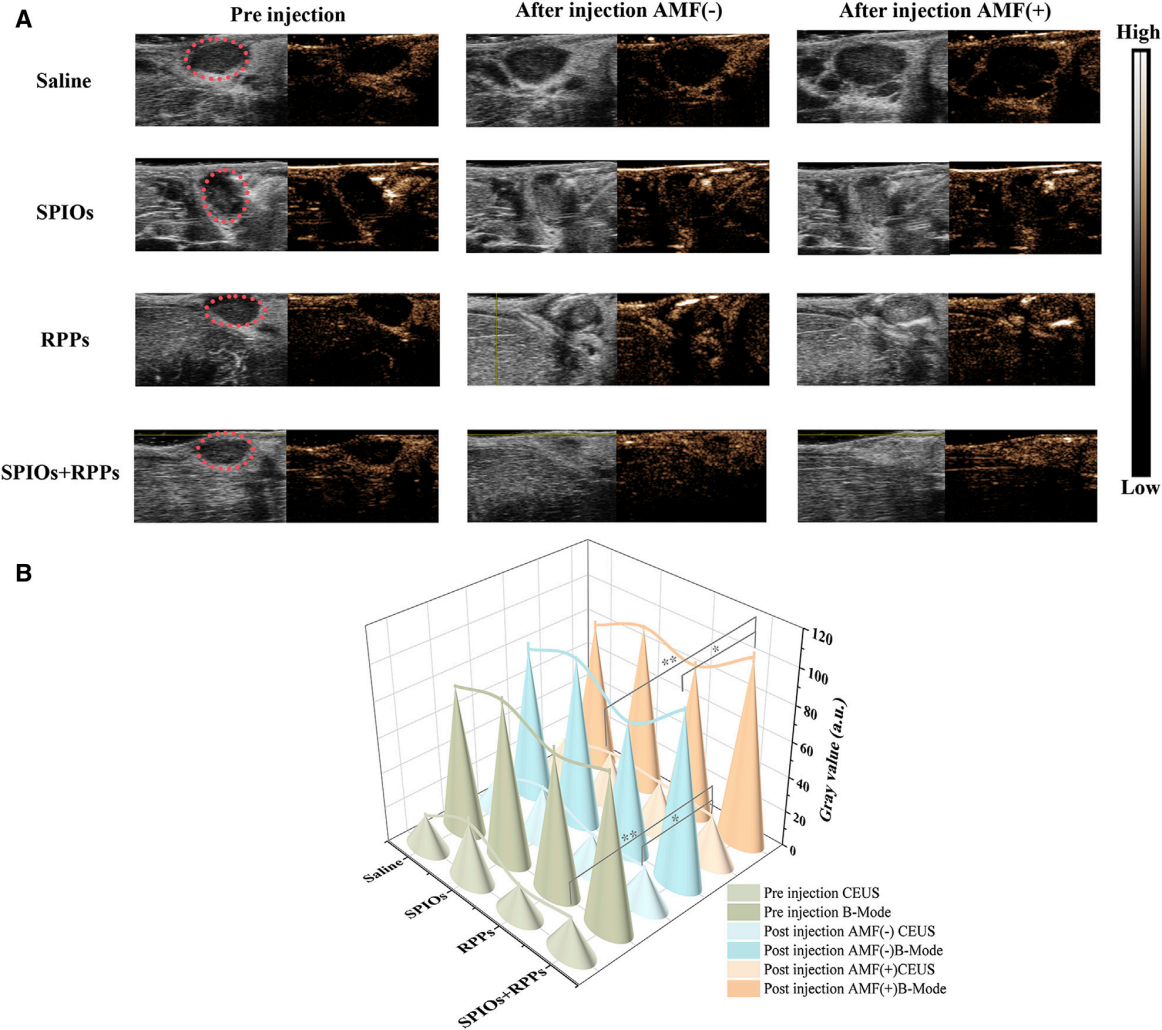
The capability of the hybrid nanosystem in SLNs ultrasound imaging. **(A)** Ultrasound imaging of popliteal fossa lymph nodes in VX2 tumor-bearing rabbits at B-mode and CEUS mode before and after the injection of saline, SPIOs, RPPs, and SPIOs + RPPs accompanied without and with magnetic heating. **(B)** Quantitative grey values of ultrasound images corresponding to images of **(A)**. (n = 3, one-way ANOVA with Tukey’s *post hoc* test, *p < 0.05, **p < 0.01)

We further assessed the imaging ability of the hybrid nanosystem as contrast agents for *in vivo* CEUS imaging via the model of VX2 tumor-bearing rabbits. As shown in Figure 4A, after exposed to AMF for magnetic heating, rabbits with the administration of SPIOs + RPPs displayed significantly improved contrast-enhanced effect in CEUS mode, while there was a slight contrast enhancement in the RPPs group. However, ultrasound signal changes were noticed in saline and SPIOs groups neither with nor without AMF stimulation. ROI of the images was quantitatively analyzed and the result was consistent with that observed in ultrasound imaging. The quantitative grey values (Figure 4B) further revealed that the ultrasound signal intensity of the SPIOs + RPPs group increased by 68.0% post magnetic heating in the CEUS mode. And the results also displayed that the ultrasound signal intensity increased by 21.6% after the injection of RPPs, while the saline group and SPIOs group showed no obvious signal intensity elevation. Currently, acoustic droplet vaporization (ADV) and optical droplet vaporization (ODV) are two main phase-transitional pathways activated by ultrasound or laser respectively. However, ADV is prone to skeletal or gas interference during sonic propagation and ODV is generally confined to the optical penetration depth (Teng et al., 2017; Wang et al., 2017). To address the drawbacks of ADV and ODV, a new magnetic field-responsive liquid–gas phase-transformation strategy was firstly proposed in our previous research, that was magnetic droplet vaporization (MDV) (Zhou et al., 2016). A significant contrast enhancement of hybrid nanosystem (SPIOs + RPPs) in CEUS mode was a powerful proof of MDV that numerous microbubbles originated from the plenitudinous phase-transition of RPPs stimulated by the heat from SPIOs under an AMF because only contrast-based ultrasound imaging modality could respond to microbubbles. And the slight contrast enhancement in RPPs group may be due to incomplete phase transition of RPPs without the thermal stimulation. *In vivo* ultrasonography investigations demonstrate the feasibility and efficiency of MDV of the hybrid nanosystem for SLNs ultrasound imaging. Ultrasonography complements SPIOs staining by allowing for an improved assessment of SLNs. Firstly, CEUS helps in providing a more accurate localization of the LNs in a non-invasive way where are not easily accessible for blue dyes or SPIOs staining. Moreover, CEUS could offer additional details on the size, shape, vascularity, and internal structure of the lymph nodes, which are crucial for assessing the extent of cancer involvement. Thirdly, ultrasonography provides real-time imaging feedback, aiding in decision-making during treatment.

### 3.4 Analysis of cells inhibition in metastatic SLN

**FIGURE 5:**
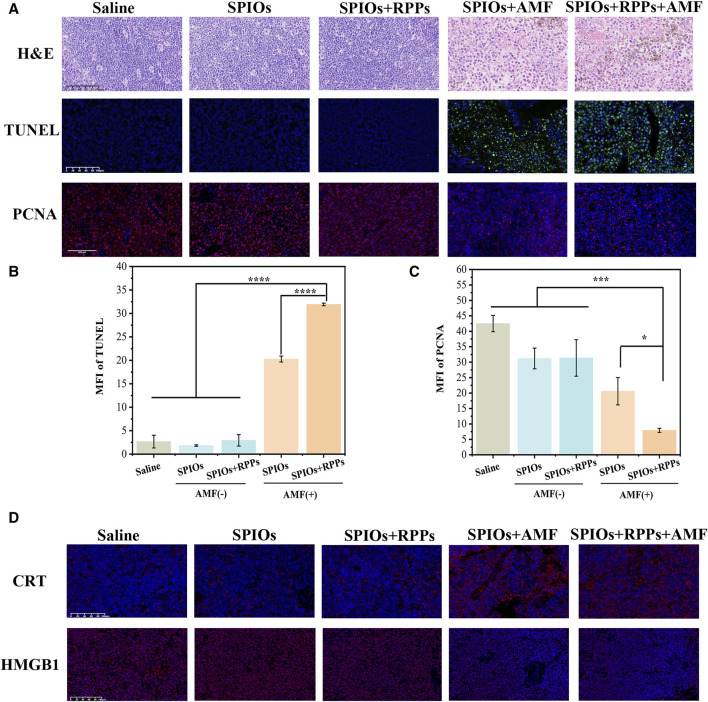
Analysis of cells inhibition in metastatic SLN. **(A)** Representative images of H&E staining, TUNEL, and PCNA immunofluorescence staining of metastatic popliteal fossa lymph nodes slices after different treatments (Scale bars = 100 μm). **(B, C)** Mean fluorescence intensity (MFI) of TUNEL and PCNA corresponding to the images of **(A)** (n = 3, one-way ANOVA with Tukey’s *post hoc* test, *p < 0.05, ***p < 0.001, ****p < 0.0001). **(D)** Immunofluorescence staining images of HMGB1/CRT in LNs tissue after the different treatment strategies (Scale bars = 100 μm).

Many researchers have focused on the application of PTT in metastatic lymph nodes (Teng et al., 2017; Wang et al., 2017), but MHT ablation of SLNs was rarely investigated. We detected therapeutic effect of MHT against metastaic lymph nodes using the hybrid nanosystem. From the H&E lymph nodes slices undergoing different treatments (Figure 5A, upper row), the SPIOs + RPPs + AMF group and SPIOs + AMF group showed severe damage, including the destruction of cell morphology, the shrinkage and rupture of cell nucelus, and apparent intercellular gaps, while the other three groups exhibited no significant structural damage. In addition, IFA of dissected lymph nodes was also performed to further verify the treatment effect of the hybrid nanosystem. From the TUNEL staining images (Figure 5A, middle row), massive apoptotic cells could be observed in SPIOs + RPPs + AMF group and SPIOs + AMF group, while the other three groups demonstrated no obvious cellular apoptosis. The PCNA staining images indicated that the proliferation of cells was more significantly inhibited in SPIOs + RPPs + AMF group and SPIOs + AMF group compared with the other three groups (Figure 5A, lower row). The quantitative analysis results of IFA were consistent with the images (Figure 5B, C). It is worthy noticed that SPIOs + RPPs + AMF group showed more obvious cellular inhibition than SPIOs + AMF group, which may be attributed to the mechanical destructive forces from cavitation effect of RPPs during MDV (such as cavitation-associated shear stress, shock waves, or microjets (Ho and Yeh, 2017; Qin et al., 2021)). The results show that SPIOs + RPPs mediated MHT can inhibit tumor cells and reduce the tumor burden effectively. Moreover, MHT is a non-invasive technique, avoiding potential complications of dissection of lymph nodes and offering a more comfortable and safer option for patients. Besides MHT ablation, the hybrid nanosystem could realize the function of ultrasound imaging, which sets the stage for the integration of diagnosis, treatment and visual monitoring in lymph node metastasis, greatly facilitating the process of theranostic.

We also verified the levels of damage-associated molecular patterns (DAMPs), the marker of immunogenic cell death (ICD) of tumor cells, in SLNs after different treatments. Surface-exposed calreticulin (CRT) and released High mobility group B1 (HMGB1) are considered two representative markers of DAMPs. CRT is exposed on the cell membrane surface, which serves as an “eat me” signal to promote phagocytosis by APCs (Abudu et al., 2019). According to the CRT staining images, the application of AMF significantly promoted the exposure of CRT compared with the no AMF stimulation groups (Figure 5D, upper row). HMGB1 as a nucleus-binding protein, is overexpressed in cancer cells, and the released HMGB1serve as a “find me” signal for APCs (Kugelberg, 2016). As shown in Figure 5D (lower row), under AMF stimulation, the content of HMGB1 decreased apparently, while the groups without AMF stimulation showed a higher content of HMGB1 in LNs tissue. The release of HMGB1 in metastatic LNs in SPIOs + AMF group and SPIOs + RPPs + AMF group are improved obviously compared with other groups. The analysis of CRT exposure and HMGB1 release in LNs tissues has proven that MHT mediated by the hybrid nanosystem could boost the impact of ICD. The increased immunogenicity of the TME paved the way for the subsequent immune response activation.

### 3.5 Immune profile of metastatic SLN

**FIGURE 6:**
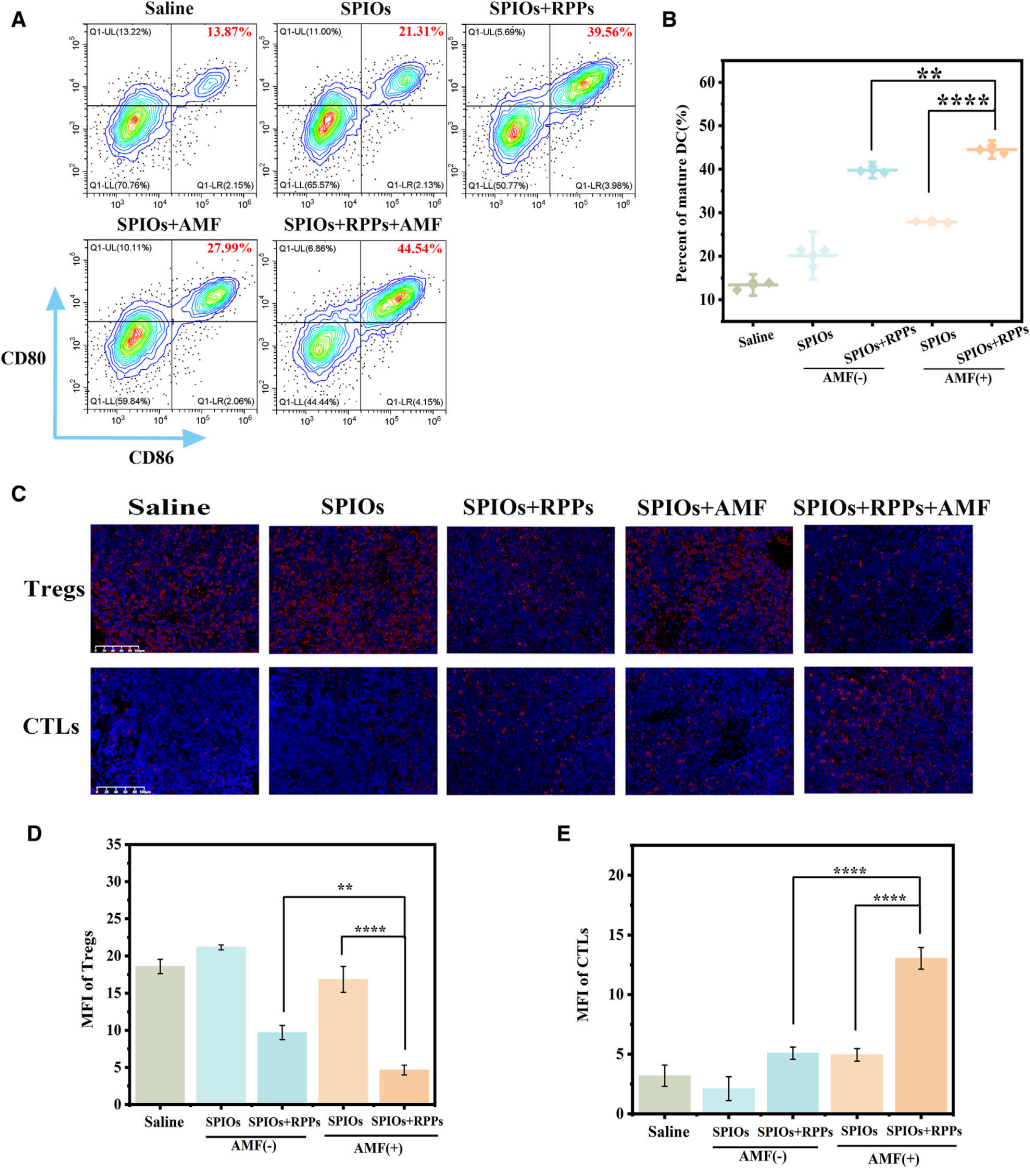
Immune profile of SLN. **(A)** Flow cytometric analysis of DC maturation in SLNs of New Zealand rabbits in different treatment groups. **(B)** The corresponding quantification of DC maturation (n = 3). **(C)** Representative images of T-regs and CTLs immunofluorescence staining of metastatic SLNs slices after different treatments (Scale bars = 100 μm). **(D, E)** Mean fluorescence intensity (MFI) of T-regs and CTLs corresponding to the images of **(C)** (n = 3, one-way ANOVA with Tukey’s *post hoc* test, *p < 0.05, ***p < 0.001, ****p < 0.0001).

We conducted immune profiles on SLNs to clarify the interaction of SPIOs + RPPs and the immune-suppressive microenvironment of metastatic lymph nodes. Flow cytometry analysis of SLNs (Figure 6A, B) showed that the SPIOs + RPPs + AMF group induced significant DC maturation (44.52%), which appeared to be much higher than that observed in the SPIOs + AMF group without R848 (27.91%) or SPIOs + RPPs group in the absence of AMF (39.80%). Furthermore, we verified the condition of T cells infiltration in SLNs via IFA. As shown in Figure 6C, there are abundant cytotoxic T cells (CTLs) and few regulatory T-cells (Tregs) in the SLNs of SPIOs + RPPs + AMF group, while the situation of the control group is on the contrary, in line with corresponding semiquantitative analysis (Figure 6D, E). These results indicate that SPIOs + RPPs mediated magnetothermal immunotherapy could effectively alleviate the immunosuppressive dilemma of SLNs. In cancer, TDLNs are under the influence of tumor-derived factors, such as IL-6, TGF-β, VEGF and prostaglandin-E2 (PGE2) (Hazelbag et al., 2002; Kim et al., 2003; Scarlett et al., 2012). As a result, DCs are suppressed and acquire an immature phenotype, and will, therefore, not properly cross-present in TDLNs (van de Ven et al., 2013). Moreover, the invasion by tumor cells can ultimately lead to the changes in composition and function of immune cells, including the enrichment of Tregs and effector T-cell exhaustion, resulting in an immune-suppressive microenvironment. And cancer cells often lose their HLA-antigens and escape immunity, as cancer antigenic peptides cannot be presented to CTLs. The effectiveness of SPIOs + RPPs in reversing the immune microenvironment may be attributed to the fact that the intense release of DAMPs after MHT, together with R848 adjuvant, created a highly immunogenic TME, efficiently stimulates the maturation of DCs to activate CTLs as well as inhibit Tregs, thus facilitate the stimulation of subsequent anti-tumor immune cascade. And MHT notably reduces the tumor burden, helping to alleviate immunosuppression at its origin. Although there is still a need to further explore the anti-tumor function and mechanism of SLNs in immunotherapy that are not yet fully understood, lower the tumor burden while preserving anti-tumor immunity in TDLNs may be a key solution to address the challenge of lymph node metastasis in cancer.

## 4 Conclusion

This study demonstrates that the hybrid nanosystem integrates the functions of SLNs staining, ultrasound imaging, and magnetic hyperthermia immunotherapy, effectively reducing the tumor burden and reversing the immune-suppressive microenvironment of metastatic SLNs in VX2 tumor-bearing rabbits with popliteal fossa lymph node metastasis. Serving as a reservoir for tumor derived antigens and the main battlefront for anti-tumor immune responses, TDLNs control a delicate balance between tumor rejection and immune tolerance. Lowering tumor burden while maintaining anti-tumor immunity in TDLNs may be an effectual therapeutic intervention to shift the balance to tumor rejection, but the timing and extent of intervention need to be further studied. In a summary, SPIOs + RPPs offers a potential as a multifunctional theranostic agent in both detection and therapy of lymphatic metastasis.

## Data Availability

The original contributions presented in the study are included in the article/Supplementary Material, further inquiries can be directed to the corresponding author.
